# Volar Distal Radioulnar Joint Dislocation Associated with Acute Median Nerve Neuropathy and a Distal Radius Fracture

**DOI:** 10.1155/2017/5674098

**Published:** 2017-08-30

**Authors:** Naser Alnusif, Sultan Aldebeyan, Rudolf Reindl

**Affiliations:** ^1^Division of Orthopaedic Surgery, McGill University, Montreal, QC, Canada; ^2^National Neuroscience Institute, King Fahad Medical City, Riyadh, Saudi Arabia

## Abstract

Volar distal radioulnar (DRUJ) dislocations are uncommon and can easily be missed. We present a rare case of an irreducible volar DRUJ dislocation associated with a distal radius fracture and acute median nerve neuropathy at the wrist. An attempt to reduce the DRUJ dislocation in the emergency department had failed. The patient was then taken to the operating room requiring a carpal tunnel release, as well as an open reduction and internal fixation of the distal radius fracture and repair of the volar distal radioulnar ligament. We also review some of the volar DRUJ case reports in the literature.

## 1. Introduction

Distal radioulnar joint dislocations are not uncommon and are usually associated with other injuries [1]. DRUJ dislocations are described based on the position of the ulna in relation to the radius with dorsal dislocation being more common [2]. Volar DRUJ dislocations are relatively uncommon and in many cases can be missed [3]. Managing such injuries starts in the emergency department by having a high index of suspicion in patients presenting with wrist injuries [4]. Once a DRUJ injury is identified, closed reduction under conscious sedation is usually attempted in the emergency department [2]. Irreducible or locked DRUJ injuries are classified as complex injuries and usually require open reduction [5]. A literature review revealed sporadic case reports of volar DRUJ dislocations [2, 5–12]. However, we present a rare case of an acute volar DRUJ dislocation associated with a distal radius fracture and acute medial nerve neuropathy at the wrist. We also review the literature of previously reported cases of volar DRUJ dislocations.

## 2. Case Presentation

A healthy right hand dominant 26-year-old male presented to the emergency department after sustaining a direct blow to his left wrist with a hockey stick while playing Lacrosse. His wrist was swollen, deformed, and positioned in supination. A complete neurological exam of his left upper extremity revealed decrease sensation over the first three digits. The vascular exam was normal with a good palpable radial pulse.

**Figure 1:**
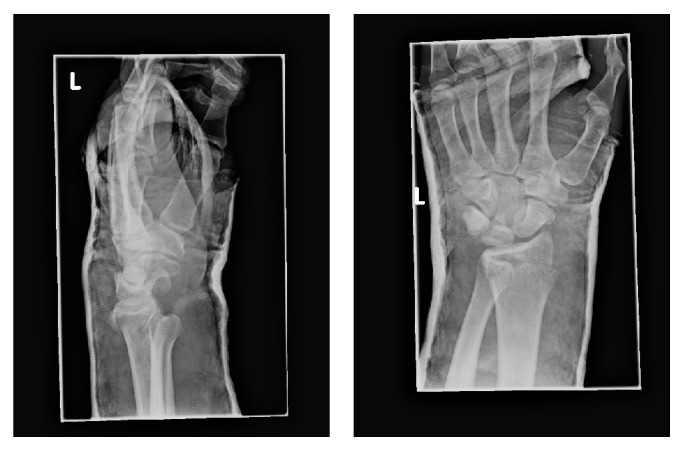
Anteroposterior and lateral X-rays of the affected wrist postimmobilization.

**Figure 2:**
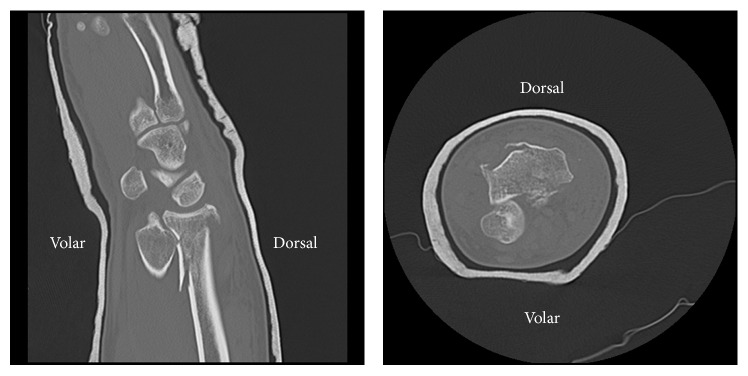
Sagittal and coronal computed tomography views showing the volar DRUJ dislocation.

Initial radiographs demonstrated a distal radius fracture associated with a severe DRUJ volar dislocation. The ulna was positioned anterior to the radius with a complete overlap of the ulna over the radius (Figure 1). Despite a trial of closed reduction and immobilization performed in the emergency department under conscious sedation, the DRUJ failed to reduce. A computed tomography was then performed for preoperative planning which confirmed the significant volar dislocation of the ulna (Figure 2). Informed consent was obtained from the patient to publish this case report.

## 3. Treatment

**Figure 3:**
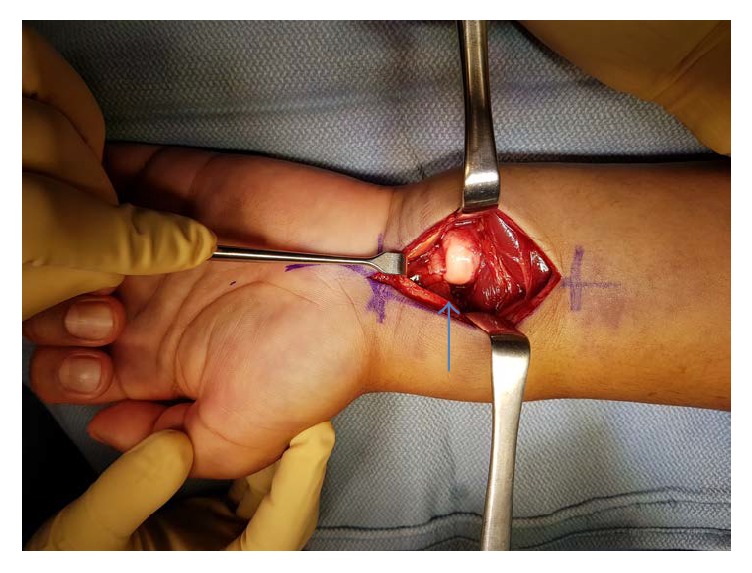
Volar surgical approach showing the volar displacement of the ulna (arrow).

**Figure 4:**
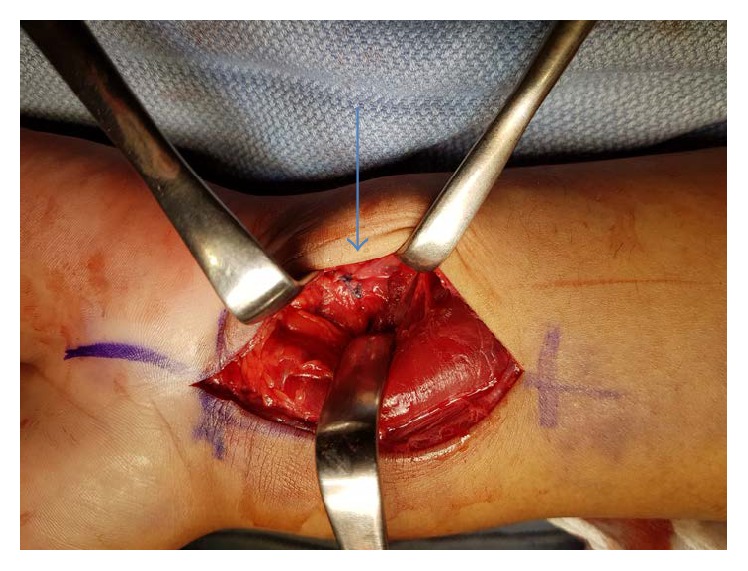
Repair of the distal radioulnar ligament (arrow).

With a diagnosis of an irreducible DRUJ dislocation associated with an acute median nerve neuropathy at the wrist and a distal radius fracture, the decision was made to urgently take the patient to the operating room. A carpal tunnel release was performed through a standard volar approach to the wrist. The ulna was dislocated and visualized volar to the distal radius compressing the median nerve (Figure 3), and, therefore, the ulna was reduced to relieve the compression off the median nerve and gain access to the distal radius fracture. Following the reduction of the radioulnar joint, the distal radius fracture was then reduced and fixed using a volar locking plate. Further assessment of the volar DRUJ ligaments revealed a volar distal radioulnar ligament mid-substance tear. The rest of the triangular fibrocartilage complex (TFCC) was intact. The DRUJ was then tested for stability and was found to be stable in pronation and unstable in supination. However, after repairing the volar distal radioulnar ligament mid-substance tear with a 2-0 Vicryl (Coated VICRYL® (polyglactin 910) Suture, Ethicon) (Figure 4), the DRUJ was very stable in full supination. The forearm was immobilized in a clamshell below elbow splint, as his DRUJ was stable on final radiographs.

## 4. Outcome and Follow-Up

**Figure 5:**
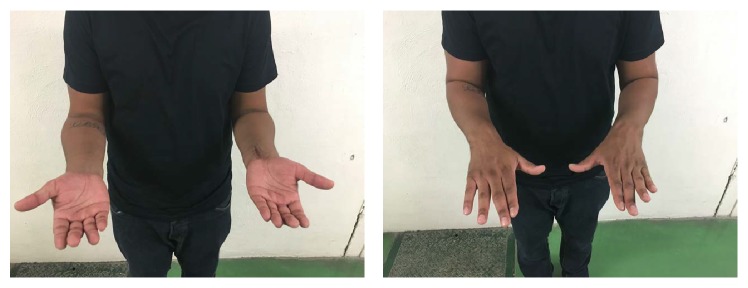
Clinical exam at 6 weeks after surgery showing full symmetrical pronation/supination.

**Figure 6:**
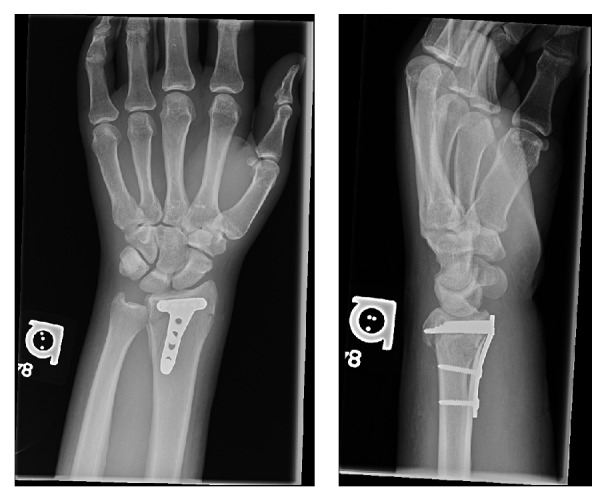
Anteroposterior and lateral X-rays of the affected wrist at the 6-week follow-up.

On the first follow-up visit at the 2-week mark, the splint was removed and the wound was completely healed. A neurological exam revealed a full recovery of the median nerve symptoms. On his 6-week follow-up visit, he was pain-free and had excellent range of motion of his left wrist. Precisely, he had full pronation/supination, which was symmetrical to his contralateral side (Figure 5). Furthermore, he was able to reach 90 degrees of extension actively; however, he had some limitation in active flexion reaching only 45 degrees. His follow-up radiographs at 6 weeks showed adequate distal radius and DRUJ alignment (Figure 6).

## 5. Discussion

Injuries to the DRUJ are frequently associated with distal radius fractures [8, 13]. Dorsal displacement of the ulna on the lateral radiograph is the common form of the injury. In contrast, volar dislocations are uncommon injuries that occur with volar impaction to the wrist with the forearm in hypersupination [8, 11]. Isolated volar dislocation of the DRUJ may be missed in up to 50% of cases [3]. Missing such injuries could lead to major sequelae and significant functional disability [14].

There are only few case reports in the literature on acute isolated volar DRUJ dislocations [7–9, 14–17]. Of these cases, only few were irreducible in the emergency department requiring surgical intervention [10–12, 18, 19]. However, none of the aforementioned case reports had acute median nerve neuropathy associated with the volar DRUJ injury. The patient presented in our study had a distal radius fracture, a locked volar DRUJ injury that failed closed reduction, and acute median nerve neuropathy at the wrist. In addition to an open reduction of both the distal radius injury and DRUJ, he also required a carpal tunnel release.

Tang et al. [11] described the DRUJ stabilizers preventing volar dislocation including the volar and dorsal distal radioulnar ligaments, joint capsule, pronator quadratus, extensor carpi ulnaris subsheath, the palmar edge of the sigmoid notch of the radius, and the TFCC being the main stabilizer. Failure of closed reduction could be due to several causes including pronator quadratus spasm [6] or impaction of the sigmoid notch of the radius as described by Garrigues and Aldridge III [18], which was the main blocking factor in the case presented in this study. In chronic cases contracted volar soft tissue could be a blocking factor to closed reduction [11].

Duryea et al. [20] described in detail the radiographic findings of DRUJ dislocations, which mandates obtaining proper true posteroanterior (PA) and lateral views of the wrist with the lateral view being the most significant in identifying DRUJ injuries. On the PA view, ulnar styloid projection should be assessed and significant radial deviation or any radioulnar overlap should raise suspicion of a DRUJ dislocation. On the true lateral view, any volar or dorsal displacement of the ulna beyond the dorsal or volar cortices of the radius should also raise suspicion of a DRUJ dislocation [20]. If the physical exam and the radiographic findings are equivocal then either an X-ray of the contralateral wrist or a computed tomography should be performed.

In summary, a high index of suspicion must be maintained when assessing patients with wrist injuries keeping a low threshold to obtain advanced imaging to assess DRUJ injuries. Detailed physical examination focusing on range of motion (supination and pronation) as well as thorough neurovascular exam to rule out acute median nerve neuropathy is crucial to prevent debilitating consequences of missed DRUJ dislocations.
